# Analysis of Hepatic Lipid Metabolism Model: Simulation and Non-Stationary Global Sensitivity Analysis

**DOI:** 10.3390/nu14234992

**Published:** 2022-11-24

**Authors:** Martina Kosić, Maja Benković, Tamara Jurina, Davor Valinger, Jasenka Gajdoš Kljusurić, Ana Jurinjak Tušek

**Affiliations:** Faculty of Food Technology and Biotechnology, University of Zagreb, 10000 Zagreb, Croatia

**Keywords:** hepatic lipid metabolism model, CellDesigner, non-stationary global sensitivity analysis, Fourier amplitude sensitivity test (FAST)

## Abstract

Lipid metabolism is a complex process and it is extremely helpful to simulate its performance with different models that explain all the biological processes that comprise it, which then enables its better understanding as well as understanding the kinetics of the process itself. Typically, kinetic parameters are obtained from a number of sources under specific experimental conditions, and they are a source of uncertainty. Sensitivity analysis is a useful technique for controlling the uncertainty of model parameters. It evaluates a model’s dependence on its input variables. In this work, hepatic lipid metabolism was mathematically simulated and analyzed. Simulations of the model were performed using different initial plasma glucose (G_B_) and plasma triacylglyceride (TAG) concentrations according to proposed menus for different meals (breakfast, lunch, snack and dinner). A non-stationary Fourier amplitude sensitivity test (FAST) was applied to analyze the effect of 78 kinetic parameters on 24 metabolite concentrations and 45 reaction rates of the biological part of the hepatic lipid metabolism model at five time points (*t*_f_ = 10, 50, 100, 250 and 500 min). This study examined the total influence of input parameter uncertainty on the variance of metabolic model predictions. The majority of the propagated uncertainty is due to the interactions of numerous factors rather than being linear from one parameter to one result. Obtained results showed differences in the model control regarding the different initial concentrations and also the changes in the model control over time. The aforementioned knowledge enables dietitians and physicians, working with patients who need to regulate fat metabolism due to illness and/or excessive body mass, to better understand the problem.

## 1. Introduction

Human diet, as well as lifestyle, influence many of the reactions and processes that occur in our bodies. Such interactions might occur at the cell, tissue, organ or organismal level. The consumption of diverse metabolites (micronutrients, macronutrients and non-nutritive dietary components) influences metabolic pathways as well as physiological homeostasis [1]. The human body is a very complicated biological system, and the same eating pattern does not have the same impact on all organisms [2]. Therefore, understanding how diet impacts an individual’s metabolism and how specific dietary patterns might enhance or harm individuals’ health are significant research aims in nutrition [3]. An efficient tool which helps solve the above problems is the use of a systems biology approach in nutrition [4]. The goal of systems biology in nutrition is to predict the mode of action of individual food components, as well as their mutual and combined action, in order to treat chronic diseases and, in certain cases, successfully prevent their occurrence [5]. According to Mc Auley [6], efficient modern nutritional research includes the most important components of systems biology: (i) bioinformatics, (ii) nutritional metabolomics, (iii) nutritional proteomics, (iv) nutritional transcriptomics, (v) nutritional genomics and (vi) computational systems biology. The results based on interconnection of mentioned approaches make it easier to understand the complex biochemical reactions and dynamic changes that occur when food is ingested into the body under certain conditions and over a certain period of time [6].

Mathematical modeling plays a very important role in the field of systems biology [6]. Mathematical models of metabolic pathways give insight into cell physiology and the evaluation of metabolic pathways used inside the cell, allowing the characterization of the metabolic behavior of the cell population [7]. Over time, mathematical models evolved, first incorporating reaction networks and stoichiometry, and later, reaction kinetics and mechanism control [8]. Today, we distinguish between: (i) constraint-based models and (iii) dynamic kinetical models [9]. Dynamic kinetic mathematical models try to represent the process inside biological systems in terms of enzyme activity and mass balance in relation to intracellular metabolites, not only in a steady state but also throughout a certain time period [10]. Such mathematical models employ differential equation systems that depict mass balance [11]. The values of intracellular fluxes and concentrations are obtained by solving the mass conservation equations numerically. These mathematical models can simulate various changes that are a based on kinetic parameters and initial concentrations [12]. Although dynamic models can be efficiently used for studying biological systems, they still have some limitations. The primary drawbacks of dynamic kinetic mathematical models are limited availability of data on intracellular molecule concentrations and a difficult technique for determining the kinetic rate law and related kinetic parameters [9].

According to Felix et al. [13], most models have numerous parameters that have to be evaluated with significant nonlinearity, which means that several solutions to the objective function can be produced during the optimization process and that the optimal combination of parameters may not be guaranteed and, therefore, the sensitivity analysis methodology needs to be used for confirming the optimal set of parameter values. Sensitivity analysis examines the impact of minor changes in nominal values of model parameters on model outputs [14]. Generally, there are two approaches: local and global sensitivity analysis. Local sensitivity analysis or one-factor-at-a-time analysis implies that parameters are varied one by one for selected small percentages, keeping other parameters constant [15]. Alternatively, global sensitivity analysis considers simulated changes of all parameter sets over a specified range [16,17]. Over the past years, different global sensitivity analysis methods such as the Moriss method [18,19,20], Sobol’s method [21,22], the Fourier amplitude sensitivity test (FAST) [23,24] and derivative-based global sensitivity measures [25,26] have been developed to study complex models. The FAST method is a well-known global sensitivity approach. The basic mechanism of the FAST method is to specify a characteristic frequency to each parameter using a search algorithm and Fourier transformation [27,28]. The FAST method then decomposes the variance of a model output into partial variances provided by individual model parameters [29]. According to Saltelli et al. [30], evaluation of sensitivity coefficients for each parameter can be conducted independently by only one simulation since all the terms in the Fourier expansion are mutually orthogonal. Based on this approach, FAST is computationally efficient for small sample sizes. To the best of our knowledge, there are limited available data on the use of the FAST method for biological model importance analysis. For example, Jurinjak Tušek et al. [31] used extended FAST for analysis of kinetic parameters of the biological part of the integrated BTEX bioremediation model. Moreover, Tušek and Kurtanjek [32] presented the use of FAST on an *E. coli* central carbon metabolism model and Jurinjak Tušek et al. [33] presented the application of FAST for analysis of the activated sludge models (ASM1, ASM2d and ASM3).

To test the importance of the parameters of the hepatic lipid metabolism model, in this work, non-stationary FAST global sensitivity analysis was applied. The goal of this work was to simulate the hepatic lipid metabolism model in order to examine how the intake of different meals with regard to the proportion of macronutrients (fats and carbohydrates) affects hepatic lipid metabolism itself and then to define the most important model parameters at five different time points to discover potential shifts in metabolic regulation.

## 2. Materials and Methods

### 2.1. Materials

#### 2.1.1. Recommendations for Menu Planning

In this work, 4 menus were designed (Table 1) that differ according to the proportion of macronutrients, with an emphasis on carbohydrates and fats.

Each menu has an energy value of 2000 kcal (±100 kcal). The USDA food composition database was used to plan meals and calculate the amount of energy and nutrients. When planning the menu, nutritional guidelines for the general population were used, as well as guidelines for the ketogenic diet. Dietary guidelines for the general population include three basic principles: variety, moderation and balance. In the case of a ketogenic diet, emphasis is placed on increased fat intake, while carbohydrate intake is extremely reduced (maximum 5% of daily energy intake) [34]. The paper presents 4 menus (Table 1): (i) menu according to the principles of a ketogenic diet (Menu 1), (ii) menu for the general population (Menu 2), and 2 residual menus, (iii) and (iv), are planned with carbohydrate and fat values that are between the first two listed menus.

#### 2.1.2. Mathematical Model of Hepatic Lipid Metabolism

In this paper, an analysis of the mathematical model of hepatic lipid metabolism was carried out. The model in the form of 24 differential equations (Table 2) including 81 parameters (Table 3) describes the metabolic response of the organism to meals with different proportions of macronutrients with a special emphasis on lipids or triglycerides. A macronutrient metabolism pathway was proposed by Pratt et al. [35]. The model includes four compartments: liver, adipose tissue, skeletal muscle and blood plasma. According to Pratt et al. [35], the liver regulates the quantities of metabolites accessible to other tissues, so each investigation of a metabolic process in the liver must also include associated tissues and organs. Nutrient distribution among tissues is controlled by blood plasma and, as such, is very important for model reliability. Moreover, adipose tissue and skeletal muscle account for 20% and 40% of the body volume and have to be taken into account in the model.

### 2.2. Methods

**Figure 1:**
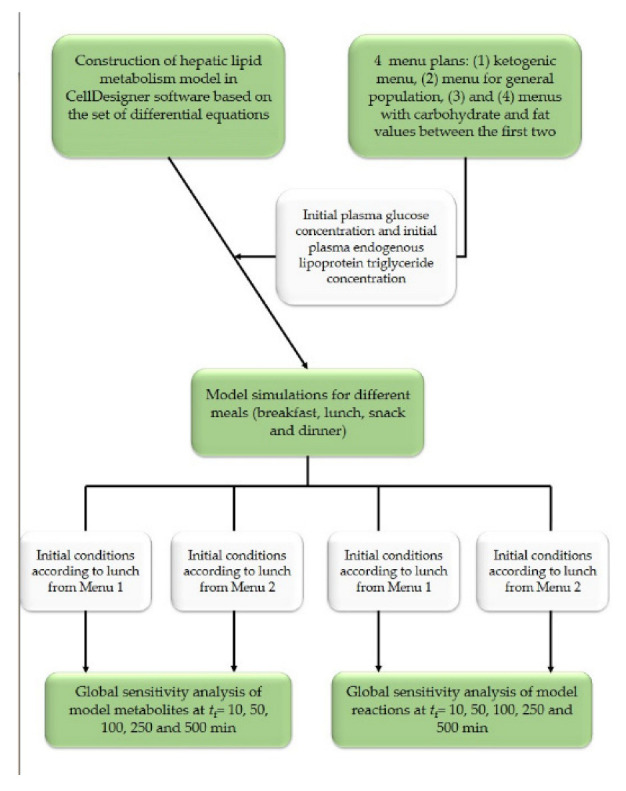
Research flowchart [35].

Schematic illustration of the experimental design and process of this study is given in Figure 1.

#### 2.2.1. Hepatic Lipid Metabolism Model Simulation

The hepatic lipid metabolism model was constructed and simulated using CellDesigner 4.4.2. Software (Systems Biology Institute (SBI), Tokyo, Japan), which allows modelling of biochemical and gene regulatory networks with a graphical interface. The simulation of hepatic lipid metabolism was performed using different initial plasma glucose concentrations (*G*_B_) and plasma endogenous lipoprotein triglyceride concentrations (*T*_LB_) based on proposed menus (Table 4). Initial values of other variables were as given by Pratt et al. [35] (Table 2).

Simulations were performed for different meals (breakfast, lunch, snack and dinner) individually. Changes in the plasma glucose concentration (*G*_B_), plasma insulin concentration (*I*), plasma non-esterified fatty acid concentration (*A*_NB_) and plasma endogenous lipoprotein triglycerides (*T*_LB_) were observed over a time period of 500 min.

#### 2.2.2. Non-Stationary Global Sensitivity Analysis

The importance of the individual parameters of the hepatic lipid metabolism model was analyzed using the Fourier amplitude sensitivity test (FAST) method belonging to a group of variance-based global sensitivity analysis methods [36]. The approach is based on the nonlinear transformation of each parameter in the multidimensional parameter space into the one-dimensional space of a single parameter, *s*, according to Equation (1):

(1) $$x_{i}=\frac{1}{2}+\frac{1}{\pi }\arcsin(\sin\left(\pi \omega_{i}s+\varphi_{i}\right)$$

where *s* is the sampling parameter with a range of *s* [−1,1], *ω_i_* are randomly chosen frequencies and *φ_i_* are randomly chosen phase angles. The phase angles are generated at random from the range *φ_i_* [−π, π], but the frequencies are integers that constitute an incommensurate set. The frequencies and phase angels are selected to provide uncorrelated parameter variations when the *s* variable is scanned throughout the limits from −1 to 1. The input variables (transformed model parameters) were sampled at frequencies ranging from 5 to 159 Hz, with the maximum frequency in the Fourier analysis set at 500 Hz. The selected range of frequencies was tested by a covariance matrix of the parameters, which showed that all parameter correlations were of order 10^−3^ or smaller, which is an indication of an almost independent parameter.

Responses of the output variable are expanded into a Fourier series by which the overall variance *D* (Equation (2)) of the output function is decomposed into summands of the squares of Fourier coefficients *A*_ω_ and *B*_ω_ (Equations (3) and (4)):

(2) $$D=2\sum_{\omega =1}^{\infty }\left(A_{\omega }^{2}+B_{\omega }^{2}\right)$$

(3) $$A_{\omega }=\frac{1}{2\pi }\cdot \int_{-\pi }^{\pi }y\left(s\right)\cdot \cos\left(\omega s\right)\cdot ds$$

(4) $$B_{\omega }=\frac{1}{2\pi }\cdot \int_{-\pi }^{\pi }y\left(s\right)\cdot \sin\left(\omega s\right)\cdot ds$$

Individual parameter sensitivity indices *S*_i_ are calculated using these Fourier coefficients. The partial sum *D_i_* of the harmonics is given by:

(5) $$D_{i}=2\cdot \sum_{p=1}^{M}A_{p\omega_{i}}^{2}+B_{p\omega_{i}}^{2}$$

(6) $$S_{i}=\frac{D_{i}}{D}$$

Wolfram Research Mathematica v. 10.0 software was used for model simulation and determining FAST sensitivities. The impacts of 78 model parameter modifications in the 2nd order of magnitude around their nominal values were evaluated simultaneously. The integration ranges of *s* from *s* = −1 to *s* = 1 were split into 2000 identical segments, each with a size of 0.001. Non-stationary sensitivity analysis was performed, and model balances for each of the 2000 parameter combinations were integrated from the starting state of signal initiation through a time span of 10, 50, 100, 250 and 500 min. The resulting set of 2000 output values is zero-order interpolated to create a continuous function *y*(*s*) of the *s* parameter. Up to the maximum frequency, the function was extended into a Fourier series. The numerical assessment of the Fourier coefficients and sensitivity coefficients was performed using zero-order interpolation. Non-stationary sensitivity analysis was applied for model metabolites and model reactions, taking into account initial conditions obtained from Menu 1 and Menu 2.

## 3. Results and Discussion

### 3.1. Meal Plans

**Figure 2:**
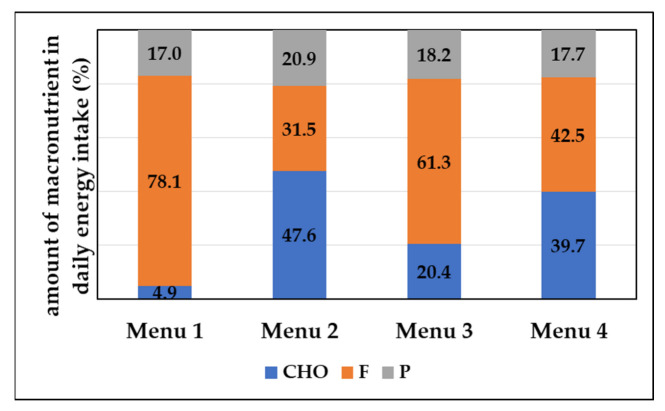
Percentage of macronutrients in daily energy intake (%). (CHO—carbohydrates, F—fats, P—proteins).

A large number of people use the ketogenic diet for the purpose of reducing body weight. The basic principle of the ketogenic diet is to drastically reduce carbohydrate intake and achieve ketosis. Ketosis is a state of the organism in which, due to the lack of carbohydrates, the formation of ketone bodies such as beta-hydroxybutyrate, acetoacetate and acetone occurs [37]. In order to achieve ketosis, it is necessary to limit carbohydrate intake to 20 to 50 g per day, and ensure the majority of daily energy intake is made up of fats [34]. As presented in Table 4 and Figure 1, in the proposed Menu 1, carbohydrate intake was 25 g and 78.1% of the total dally energy intake corresponded to fats. Menu 2 was designed according to dietary guidelines for the general population with regard to the proportion of macronutrients. Accordingly, the proportion of fat should be between 20–35% of the total daily energy intake [38] and the proportion of carbohydrates should be between 45–60% of the total daily energy intake [39]. Accordingly, with an energy intake of 2000 kcal, carbohydrate intake should be between 225 and 300 g, and fat intake between 44.4 and 77.8 g. Menu 2 included 238.5 g of carbohydrates per day that contributed to 47.6% of daily energy intake and 70.2 g of fats per day contributing to 31.5% of daily energy intake (Table 5 and Figure 2). The proportion of saturated fatty acids (SFA) ranged from 1.2% in the daily energy supply of Menu 2 (average menu for the general population) to 16.2% for Menu 1 (keto diet menu). The other two menus (Menu 3 and Menu 4) have carbohydrate and fat proportions between the first two listed dietary patterns in order to further examine the impact of different carbohydrate and fat intakes on fat metabolism. Menu 3 included 101.9 g of carbohydrates per day that contribute to 20.4% of daily energy intake and 136.0 g of fats per day contributing to 61.3% of daily energy intake, while Menu 4 included 200 g of carbohydrates per day that contribute to 39.7% of daily energy intake and 95.1 g of fats per day contributing to 42.5% of daily energy intake (Table 5 and Figure 2).

### 3.2. Hepatic Lipid Metabolism Construction and Simulation in CellDesigner

**Figure 3:**
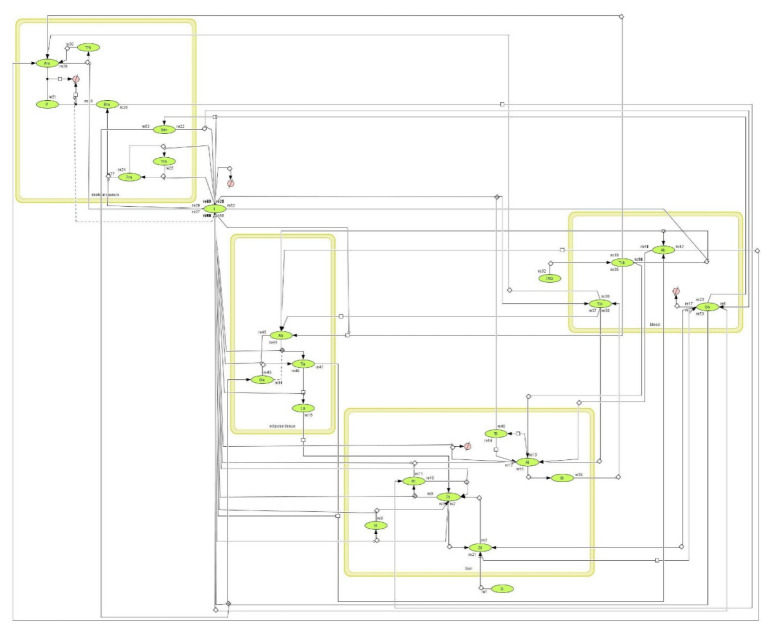
CellDesigner model of hepatic lipid metabolism model.

CellDesigner is widely used for biochemical modeling due to its straightforward visualization and presentation of the logic and dynamics of complicated processes inherent in most metabolic pathways [40]. In this work, CellDesigner was used for hepatic lipid model simulation and analysis. As described by Funahaski et al. [41], CellDesigner is a tool for modeling gene regulatory and metabolic networks that enables users to quickly design such networks through the use of well-defined and detailed graphical representations. The CellDesigner model of lipid metabolism constructed based on the list of balances given in Table 1 and using parameter values given in Table 2 is presented in Figure 3.

**Figure 4:**
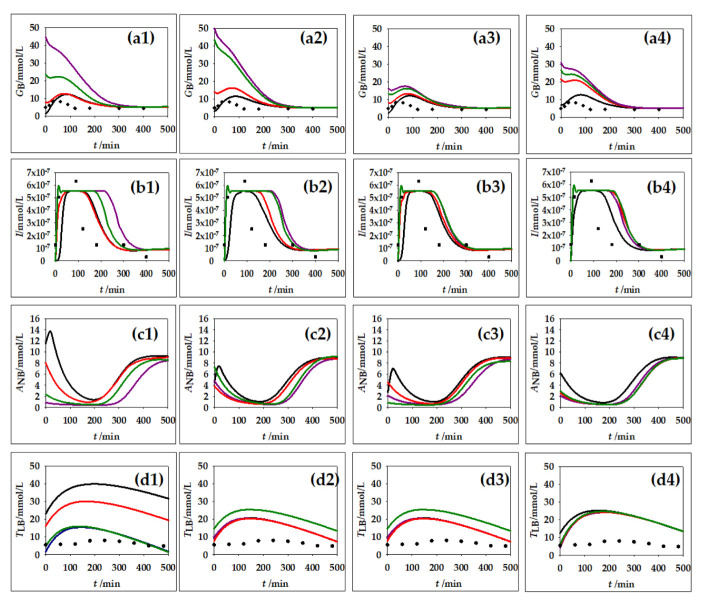
Results of hepatic lipid metabolism model simulations in CellDesigner software. (**a1**–**a4**) plasma glucose concentration, (**b1**–**b4**) plasma insulin concentration, (**c1**–**c4**) plasma non-esterified fatty acid concentration, (**d1**–**d4**) plasma endogenous lipoprotein triglycerides. Initial concentration for different meals (1) breakfast, (2) lunch, (3) snack and (4) dinner according to different menus (**⎯**) Menu 1, (**⎯**) Menu 2, (**⎯**) Menu 3, (**⎯**) Menu 4. (♦) experimental data for plasma glucose concentration from Yoshizame et al. [44], (▪) experimental data for plasma insulin concentration from Yoshizame et al. [44] (•) experimental data for plasma concentration of triacylglycerol from Sarabhai et al. [45].

Lipids make up a very important part of human nutrition. Triglycerides are mostly ingested through food. The main places of triglyceride storage are within fat cells. Inside the fat cells, the synthesis and breakdown of triglycerides and, if necessary, the mobilization of triglycerides to other tissues are possible [42]. For this reason, changes in lipid metabolism can lead to the development of many disorders and diseases in the body. As part of this work, four simulations were carried out with regard to different concentrations of blood glucose and triglycerides. The of carbohydrate and fat contents from the menu shown in Table 4 were used for calculation of the initial quantities of glucose and triglycerides and the calculation was made individually for each meal. In addition to the changes in the concentration of plasma glucose concentration, plasma insulin, plasma non-esterified fatty acids and plasma endogenous lipoprotein triglycerides were monitored over a period of 500 min. Obtained results are presented in Figure 4.

According to Cruz et al. [43], after food intake, there is a glucose increase in the blood, given that glucose is absorbed in the intestines, from where it spreads through the blood to other peripheral tissues. Blood glucose concentration in healthy individuals should be between 4.9 and 6.9 mmol/L [43]. Figure 4(a1–a4) clearly shows an initial increase in blood glucose depending on the initial concentration of glucose in a particular meal. The concentration stabilizes at around 240 min. It can also be seen that Breakfast 2 (Figure 4(a1)), which is extremely rich in carbohydrates, i.e., glucose (Table 4), leads to a fast increase in blood glucose, after which the concentration decreases. The same can be observed in Figure 4(a2) with Lunch 2 and Lunch 4.

Insulin is a hormone whose action is influenced by glucose. After food intake, insulin is secreted from the β-cells of the pancreas. The role of insulin is to distribute glucose from the blood to peripheral tissues [46]. The insulin secretion curve has two characteristic parts: the first part, which is characterized by a rapid rise in insulin concentration after which it disappears after several minutes, and the second part, which lasts longer, that stabilizes and slowly decreases insulin concentration [47]. Such nature of insulin behavior can also be observed in Figure 4(b1–b4). Immediately after food intake, there is a large increase in the concentration of insulin in the blood, then stabilization of the concentration occurs and, finally, a slight decrease in the concentration can be observed. In Figure 3, it can also be observed that a higher concentration of glucose in the blood causes a longer action of insulin before the concentration decreases and stabilizes.

The influence of dietary triglycerides on insulin secretion was studied by Bermudez et al. [48]. According to their research, monounsaturated fatty acids such as oleic acid have a positive influence on insulin sensitivity, and saturated fatty acids such as palmitic acid have a negative influence. In addition to the concentration of triglycerides in an individual meal, the type of fatty acids from which the triglycerides themselves are made is also important. Consistency with experimental data from the research of Cruz et al. [43] was also confirmed in the example of non-esterified fatty acids (A_NB_). Initially, in response to food intake and insulin action, there is a decrease in A_NB_ concentration. Insulin inhibits the action of the enzyme lipase, which is responsible for the formation of A_NB_ [49]. Figure 4(c1–c4) clearly shows that after food intake, there is a decrease in A_NB_ concentration. By comparing subfigures in Figure 4(b1–b4), which present changes in insulin concentration and changes in A_NB_ concentration, the mentioned dependence of A_NB_ concentration on insulin concentration can be observed. When the insulin concentration decreases, the A_NB_ concentration increases and vice versa. This is clearly visible in the examples of Breakfast 1, Lunch 1 and Snack 1.

The largest proportion of fat that we consume in food refers to triglycerides (TAG). Triglycerides must be broken down to fatty acids to become available for absorption in the intestinal epithelium. After that, triglycerides are again resynthesized in the intestinal epithelium, and are then incorporated into lipoprotein particles called chylomicrons. Then, chylomicrons are released into the lymph and then into the blood, where they transport triglycerides to peripheral tissues [42]. Figure 4(d1–d4) shows the behavior of triglycerides in the blood during the 500 min following a meal. This behavior is in accordance with the stated theoretical facts; first, there is a slight increase in the concentration of triglycerides, which, after some time, begins to decrease and later returns to the initial value. The concentration of triglycerides after a meal is directly dependent on the amount of ingested fat [50]. The biggest increase in triglyceride concentration is caused by meals with the highest TAG content. The higher the proportion of triglycerides in the meal, the longer it takes for the concentration to return to the initial value.

The hepatic lipid metabolism model simulation results for CellDesigner were compared with available experimental data from the literature. The simulation results for glucose and insulin dynamic change were compared with the data given by Yoshizame et al. [44], where the time course of blood glucose and insulin levels were dynamically measured after ingestion of 25 g of trehalose or glucose among 20 participants in the study. Furthermore, simulation results for the plasma endogenous lipoprotein triglycerides were compared with the experimental data presented by Sarabhai et al. [45], where plasma concentration of triacylglycerol was measured among 16 volunteers after receiving single meals containing safflower oil, palm oil or other natural vehicles for stabilizing and storing biological lipophilic compounds. The obtained simulation results follow the trend of the experimental data well, especially for the glucose concentration. The biggest difference between the used experimental data and model simulation results was noticed for the plasma endogenous lipoprotein triglyceride concentration change, due to different meal compositions used for obtaining experimental data and the model simulation. However, based on the presented results, it can be concluded that mathematical modeling can be efficiently used for the prediction of metabolite profiles.

### 3.3. Non-Stationary Global Sensitivity Analysis

Mathematical models make it possible to integrate information collected from different sources using common mathematical methods. By increasing the availability of information about the metabolic activity of an organism through the application of advanced molecular techniques, the complexity of the proposed mathematical models also increases [51]. Mathematical models of biological systems are most often derived in the form of differential equations that describe the changes in a single variable over time [52]. When developing models of biological systems, the biggest challenge is the choice, that is, the estimation of model parameter values (maximum reaction rates, saturation constants, etc.). Due to still-existing experimental limitations, it is sometimes impossible to estimate the real value of a particular model parameter, so the accuracy of the selected model parameter value is often questionable. This problem is particularly related to parameters that describe the kinetic expressions of enzyme-catalyzed reactions. Specifically, the estimation of parameter values of kinetic models of enzyme-catalyzed reactions is usually carried out on the basis of data collected in in vitro experiments, and often, in vitro conditions do not simulate in vivo conditions with complete accuracy [14]. In order to examine the influence of a change in the value of a model parameter on the selected output variables, parametric sensitivity analysis techniques are applied. The analysis of local parametric sensitivity is based on small changes in the value of an individual parameter, while the values of other parameters are constant [53]. On the other hand, the analysis of global parametric sensitivity includes the analysis of the impact of changes in the values of all model parameters simultaneously [31].

In this work, global parametric sensitivity analysis was performed using Fourier analysis (Fourier amplitude sensitivity test, FAST). The FAST method assumes that each parameter in the mathematical model is statistically independent of other parameters. Each parameter is varied at different frequencies and the output amplitudes are measured [54]. Global sensitivity coefficients were estimated for all 24 model variables and 45 reactions for two different initial concentrations of glucose and triglycerides according to designed menus for Lunches 1 and 2. Global sensitivity coefficients were estimated at five different time intervals (*t*_f_ = 10, 50, 100, 200 and 500 min) to define the control change over time. The global sensitivity coefficients are marked with colors: green indicating low values of sensitivity coefficients, and red indicating high values of global sensitivity coefficients based on the data distribution at the percentile curve.

#### 3.3.1. Global Sensitivities of the Model Metabolites

**Figure 5:**
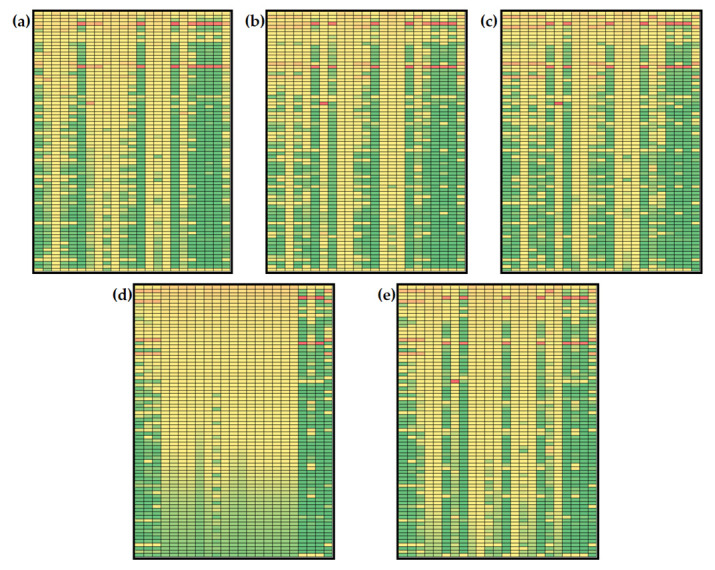
Global sensitivities of hepatic lipid model metabolites (initial conditions for simulations according to Menu 1): (**a**) *t*_f_ = 10 min, (**b**) *t*_f_ = 50 min, (**c**) *t*_f_ = 100 min, (**d**) *t*_f_ = 250 min, (**e**) *t*_f_ = 500 min. Rows: *β*_G_, *β*_6_, *β*_f_, *β*_l_, *β*_m_, *µ*_AMP_, *µ*_e_, *µ*_s_, *µ*_1_, *µ*_2_, *µ*_3_, *µ*_4_,*c*_0_, *c*_c_, *d*_BA_, *k*_10_, *k*_11_, *k*_12_, *k*_13_, *k*_14_, *k*_22_, *k*_5_, *k*_6_, *k*_6l_, *k*_6p_, *k*_7_, *k*_8_, *k*_9_, *k*_9a_, *k*_a_, *k*_aa_, *k*_ai_, *k*_al_, *k*_ba_, *k*_bl_, *k*_bm_, *k*_cl_, *k*_cm_, *k*_d_, *k*_dl_, *k*_dy_, *k*_ft_, *k*_ga_, *k*_gi_, *k*_gl_, *k*_gl2_, *k*_gm_, *k*_gm2_, *k*_gp_, *k*_lp_, *k*_LG_, *k*_LH_, *k*_MH_, *k*_na_, *k*_p_, *k*_p6_, *k*_pp_, *k*_r_, *k*_re_, *k*_t_, *k*_yl_, *k*_ym_, *l*_max_, *m*_max_, *v*_min_, *v*_10,_ *v*_12_, *v*_6_, *v*_8_, *v*_9_, *v*_LG_, *v*_LH_, *v*_MH_, *y*_0_, *α*_G_, *α*_F_, *m*_s_, *m*_e_. Columns: *I*, *G*_L_, *Y*_L_, *P*_L_, *R*_L_, *A*_L_, *S*_L_, *T*_L_, *G*_M_, *Y*_M_, *P*_M_, *R*_M_, *A*_M_, *T*_M_, *P*, *T*_A_, *A*_A_, *L*_A_, *G*_A_, *T*_CB_, *A*_NB_, *T*_LB_, *G*_B_. The global sensitivity coefficients are marked with colors: green indicating low values of sensitivity coefficients, and red indicating high values of global sensitivity coefficients based on the data distribution at the percentile curve.

**Figure 6:**
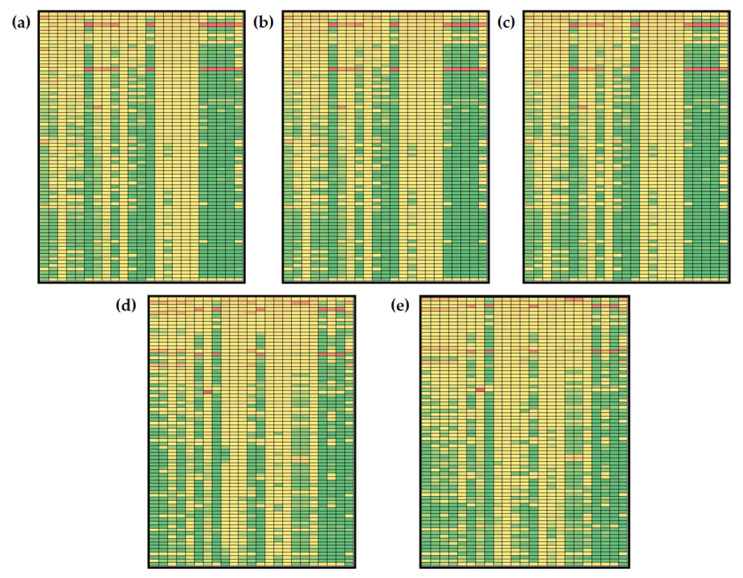
Global sensitivities of hepatic lipid model metabolites (initial conditions for simulations according to Menu 2): (**a**) *t*_f_ = 10 min, (**b**) *t*_f_ = 50 min, (**c**) *t*_f_ = 100 min, (**d**) *t*_f_ = 250 min, (**e**) *t*_f_ = 500 min. Rows: *β*_G_, *β*_6_, *β*_f_, *β*_l_, *β*_m_, *µ*_AMP_, *µ*_e_, *µ*_s_, *µ*_1_, *µ*_2_, *µ*_3_, *µ*_4_,*c*_0_, *c*_c_, *d*_BA_, *k*_10_, *k*_11_, *k*_12_, *k*_13_, *k*_14_, *k*_22_, *k*_5_, *k*_6_, *k*_6l_, *k*_6p_, *k*_7_, *k*_8_, *k*_9_, *k*_9a_, *k*_a_, *k*_aa_, *k*_ai_, *k*_al_, *k*_ba_, *k*_bl_, *k*_bm_, *k*_cl_, *k*_cm_, *k*_d_, *k*_dl_, *k*_dy_, *k*_ft_, *k*_ga_, *k*_gi_, *k*_gl_, *k*_gl2_, *k*_gm_, *k*_gm2_, *k*_gp_, *k*_lp_, *k*_LG_, *k*_LH_, *k*_MH_, *k*_na_, *k*_p_, *k*_p6_, *k*_pp_, *k*_r_, *k*_re_, *k*_t_, *k*_yl_, *k*_ym_, *l*_max_, *m*_max_, *v*_min_, *v*_10,_ *v*_12_, *v*_6_, *v*_8_, *v*_9_, *v*_LG_, *v*_LH_, *v*_MH_, *y*_0_, *α*_G_, *α*_F_, *m*_s_, *m*_e_. Columns: *I*, *G*_L_, *Y*_L_, *P*_L_, *R*_L_, *A*_L_, *S*_L_, *T*_L_, *G*_M_, *Y*_M_, *P*_M_, *R*_M_, *A*_M_, *T*_M_, *P*, *T*_A_, *A*_A_, *L*_A_, *G*_A_, *T*_CB_, *A*_NB_, *T*_LB_, *G*_B_. The global sensitivity coefficients are marked with colors: green indicating low values of sensitivity coefficients, and red indicating high values of global sensitivity coefficients based on the data distribution at the percentile curve.

Based on the results of metabolites’ global sensitivities for the model simulation according to initial concentrations from Menu 1 and Menu 2 (Figure 5 and Figure 6), it can be noticed that estimated values of sensitivities are in the range for 1.750 × 10^−5^ to 0.895 for Menu 1 and in the range from 6.000 × 10^−5^ to 0.986 for Menu 2. Furthermore, changes in the model control over time can be observed. Throughout time, model parameters’ sensitivity coefficients change with different dynamics.

Some sensitivity coefficients’ values increase over time, while some decrease. Furthermore, some similarities can be observed. Numerically, the largest values of sensitivity coefficients (values over 0.80) were obtained for *β*_L_ (liver glycogenolysis rate) and *k*_11_ (basal insulin secretion rate) for both combinations of initial conditions. *β*_L_ is a kinetic constant included in the description of liver glycogen balance. It describes the rate of liver glycogen release and conversion to glucose-6-phosphate inhibited by insulin. *k*_11_ is included in insulin balance and it describes the glucose-stimulated insulin production. Using initial concentrations based on Menu 1 and Menu 2 for model simulation, high values of sensitivity coefficients were calculated for free fatty acid concentration in the liver (*A*_L_), free fatty acid concentration in muscle (*A*_M_), free fatty acid concentration in adipose tissue (*A*_A_), glucose concentration in adipose (*G*_A_), triglycerides in liver secretion pool (*T*_L_), triglyceride concentration in blood plasma (*T*_CB_), free fatty acid concentration in plasma (*A*_NB_) and endogenous triglyceride concentration in plasma (*T*_LB_) at *t*_f_ = 10, 50, 100 and 250 min. At *t*_f_ = 500 min, changes in *β*_L_ and *k*_11_ values mostly affected *T*_CB_ and *T*_LB_ concentrations for initial conditions based on the keto diet menu (Figure 5e) and additional *T*_L_ for initial conditions based on the general population menu (Figure 6e). It is also important to mention that results show global sensitivity coefficients for *Y*_M_ and *T*_M_ over the 50th percentile of calculated coefficients (marked yellow) for all parameters at *t*_f_ = 10, 50, 100 and 250 min for Menu 1 (Figure 5a–d). For Menu 2, global sensitivity coefficients for *Y*_M_, *T*_M_ and *T*_A_ were over the 50th percentile of calculated coefficients for all analyzed model parameters at all five time points, indicating that those variables are the most sensitive part of the model (Figure 6a–e).

Changes in the concentration of plasma glucose, plasma insulin, plasma non-esterified fatty acids and plasma endogenous lipoprotein triglycerides were analyzed in the previous section, so it was considered important to discuss the sensitivities of the selected model variables. As described before, *A*_NB_ and *T*_LB_ were mostly sensitive to variations in *β*_L_ and *k*_11_ values for both menus at all five analyzed time points. Furthermore, changes in control were noticed for *G*_B_ and *I*. For Menu 1 (keto diet), *G*_B_ concentration is mostly sensitive to *β*_L_, *k*_11_ and k_aa_ (rate of adipose free fatty acid esterification to triglycerides) values at *t*_f_ = 10 min (Figure 5a). Prolonging the simulation time to *t*_f_ = 50, 100, 250 and 500 min, results showed that *G*_B_ concentration is mostly sensitive to *β*_6_ (rate of liver de novo lipogenesis form pyruvate), *β*_M_ (muscle glycogenolysis rate), *k*_10_ (affinity for hydrolysis of triglycerides to secretory pool) and *k*_14_ (basal very low-density lipoprotein 1 secretion fraction) (Figure 5b,e). A similar observation was noted for *I* concentration: at *t*_f_ = 10 min (Figure 5a), the *I* concentration is mostly sensitive to *β*_6_ (rate of liver de novo lipogenesis from pyruvate), *β*_L_, *β*_M_ (muscle glycogenolysis rate), *k*_10_ (affinity for hydrolysis of triglycerides to secretory pool), *k*_11_ and *k*_14_ (basal very low-density lipoprotein 1 secretion fraction), while at *t*_f_ = 50, 100, 250 and 500 min, results showed that the *I* concentration was most sensitive to *β*_6_, *β*_M_, *k*_10_ and *k*_14_ (Figure 5b,e). Moreover, for Menu 2 (general population diet) at *t*_f_ = 10 min (Figure 6a), *G*_B_ concentration is mostly sensitive to *β*_L_, and *k*_11_ values, but prolonging the simulation time to *t*_f_ = 50, 100, 250 and 500 min, results showed that *G*_B_ concentration becomes mostly sensitive to *β*_6_, *β*_M_, *k*_10_ and *k*_14_ (Figure 6b,e). At *t*_f_ = 10 min, the *I* concentration is mostly sensitive to *β*_6_, *µ*_3_ (rate of muscle glucose-6-phospahe usage) and *k*_cm_ (muscle free fatty acid uptake of chylomicron triglycerides) (Figure 6a), while results showed that the *I* concentration was the most sensitive to *β*_6_, *β*_M_, *k*_10_ and *k*_14_ at *t*_f_ = 50, 100, 250 and 500 min (Figure 5b,e).

#### 3.3.2. Global Sensitivities of the Model Reactions

**Figure 7:**
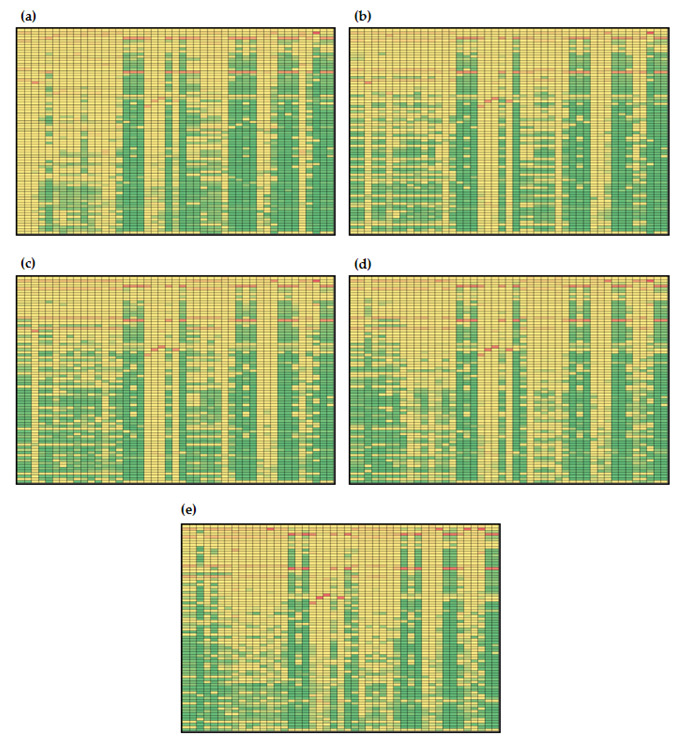
Global sensitivities of hepatic lipid model reactions (initial conditions for simulations according to Menu 1): (**a**) *t*_f_ = 10 min, (**b**) *t*_f_ = 50 min, (**c**) *t*_f_ = 100 min, (**d**) *t*_f_ = 250 min, (**e**) *t*_f_ = 500 min. Rows: *β*_G_, *β*_6_, *β*_f_, *β*_l_, *β*_m_, *µ*_AMP_, *µ*_e_, *µ*_s_, *µ*_1_, *µ*_2_, *µ*_3_, *µ*_4_,*c*_0_, *c*_c_, *d*_BA_, *k*_10_, *k*_11_, *k*_12_, *k*_13_, *k*_14_, *k*_22_, *k*_5_, *k*_6_, *k*_6l_, *k*_6p_, *k*_7_, *k*_8_, *k*_9_, *k*_9a_, *k*_a_, *k*_aa_, *k*_ai_, *k*_al_, *k*_ba_, *k*_bl_, *k*_bm_, *k*_cl_, *k*_cm_, *k*_d_, *k*_dl_, *k*_dy_, *k*_ft_, *k*_ga_, *k*_gi_, *k*_gl_, *k*_gl2_, *k*_gm_, *k*_gm2_, *k*_gp_, *k*_lp_, *k*_LG_, *k*_LH_, *k*_MH_, *k*_na_, *k*_p_, *k*_p6_, *k*_pp_, *k*_r_, *k*_re_, *k*_t_, *k*_yl_, *k*_ym_, *l*_max_, *m*_max_, *v*_min_, *v*_10,_ *v*_12_, *v*_6_, *v*_8_, *v*_9_, *v*_LG_, *v*_LH_, *v*_MH_, *y*_0_, *α*_G_, *α*_F_, *m*_s_, *m*_e_. Columns: *I*, *G*_L_, *Y*_L_, *P*_L_, *R*_L_, *A*_L_, *S*_L_, *T*_L_, *G*_M_, *Y*_M_, *P*_M_, *R*_M_, *A*_M_, *T*_M_, *P*, *T*_A_, *A*_A_, *L*_A_, *G*_A_, *T*_CB_, *A*_NB_, *T*_LB_, *G*_B_. The global sensitivity coefficients are marked with colors: green indicating low values of sensitivity coefficients, and red indicating high values of global sensitivity coefficients based on the data distribution at the percentile curve.

**Figure 8:**
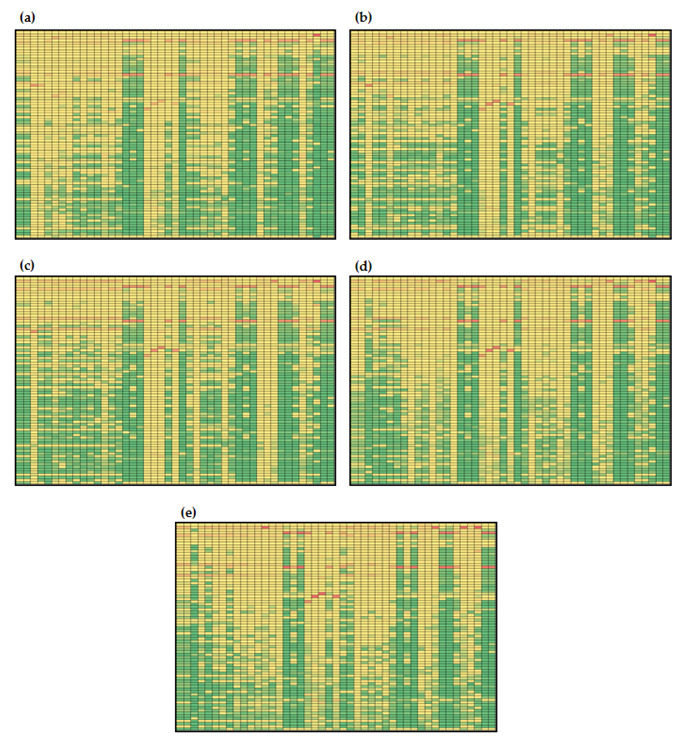
Global sensitivities of hepatic lipid model reactions (initial conditions for simulations according to Menu 2): (**a**) *t*_f_ = 10 min, (**b**) *t*_f_ = 50 min, (**c**) *t*_f_ = 100 min, (**d**) *t*_f_ = 250 min, (**e**) *t*_f_ = 500 min. Rows: *β*_G_, *β*_6_, *β*_f_, *β*_l_, *β*_m_, *µ*_AMP_, *µ*_e_, *µ*_s_, *µ*_1_, *µ*_2_, *µ*_3_, *µ*_4_,*c*_0_, *c*_c_, *d*_BA_, *k*_10_, *k*_11_, *k*_12_, *k*_13_, *k*_14_, *k*_22_, *k*_5_, *k*_6_, *k*_6l_, *k*_6p_, *k*_7_, *k*_8_, *k*_9_, *k*_9a_, *k*_a_, *k*_aa_, *k*_ai_, *k*_al_, *k*_ba_, *k*_bl_, *k*_bm_, *k*_cl_, *k*_cm_, *k*_d_, *k*_dl_, *k*_dy_, *k*_ft_, *k*_ga_, *k*_gi_, *k*_gl_, *k*_gl2_, *k*_gm_, *k*_gm2_, *k*_gp_, *k*_lp_, *k*_LG_, *k*_LH_, *k*_MH_, *k*_na_, *k*_p_, *k*_p6_, *k*_pp_, *k*_r_, *k*_re_, *k*_t_, *k*_yl_, *k*_ym_, *l*_max_, *m*_max_, *v*_min_, *v*_10,_ *v*_12_, *v*_6_, *v*_8_, *v*_9_, *v*_LG_, *v*_LH_, *v*_MH_, *y*_0_, *α*_G_, *α*_F_, *m*_s_, *m*_e_. Columns: *I*, *G*_L_, *Y*_L_, *P*_L_, *R*_L_, *A*_L_, *S*_L_, *T*_L_, *G*_M_, *Y*_M_, *P*_M_, *R*_M_, *A*_M_, *T*_M_, *P*, *T*_A_, *A*_A_, *L*_A_, *G*_A_, *T*_CB_, *A*_NB_, *T*_LB_, *G*_B_. The global sensitivity coefficients are marked with colors: green indicating low values of sensitivity coefficients, and red indicating high values of global sensitivity coefficients based on the data distribution at the percentile curve.

Global sensitivities of hepatic lipid metabolism models are given in Figure 6 and Figure 7. It can be noticed that estimated values of global sensitivities are in the range from 5.530 × 10^−7^ to 0.986 for Menu 1 (Figure 7) and in the range from 6.000 × 10^−7^ to 0.986 for Menu 2 (Figure 8). As for the model metabolites, model reaction changes in the model control were observed over time. Similar to the model metabolites, numerically, the largest values of sensitivity coefficients (values over 0.75) were obtained for *β*_L_ and *k*_11_ and, additionally, for *β*_6_ for both initial conditions (Figure 7 and Figure 8). Results showed that the largest global sensitivities were obtained for variations in *β*_L_, *k*_11_ and *β*_6_ values for both menus for the following reactions: (i) *v*_16_ (uptake of exogenous plasma triglycerides into liver free fatty acids), (ii) *v*_17_ (free fatty acid transport from plasma to liver), (iii) *v*_18_ (free fatty acid synthesis form plasma endogenous triglycerides), (iv) *v*_24_ (rate of triglyceride release into plasma), (v) *v*_32_ (uptake of exogenous plasma triglycerides into muscle free fatty acids), (vi) *v*_34_ (rate of endogenous triglyceride release into plasma), (vii) *v*_38_ (uptake of plasma exogenous triglycerides by adipose tissue), (viii) *v*_39_ (uptake of plasma endogenous triglycerides into adipose free fatty acids), (ix) *v*_44_ (rate of complete uptake of plasma exogenous triglycerides by adipose tissue) and (x) *v*_45_ (adipose tissue uptake of exogenous triglycerides from plasma). Additionally, a sensitivity coefficient over 0.75 was obtained for the influence of *β*_6_ on v_43_ (fat input form the diet). The obtained result confirms the statement by Pratt et al. [35] where they describe that under conditions of heightened insulin, the liver accumulates glucose in form of glycogen (glycogenesis) for later use, and during times of low insulin, the liver decomposes glycogen to glucose (glycogenolysis).

Furthermore, it can also be noticed that for simulations with initial conditions according to Menu 1 (Figure 7), only global sensitivity coefficients for *v*_19_ were over the 50th percentile of calculated coefficients (marked yellow) for all parameters at *t*_f_ = 100 min (Figure 7c). With described initial conditions at *t*_f_ = 100 min, the highest sensitivities for reaction *v*_19_ were estimated for *β*_L_ (0.617), *k*_a_ (0.603) and *β*_M_ (0.157), indicating that triglyceride storage conversion to free fatty acids in the liver mostly depends on the release of liver glycogen, uptake of plasma triglycerides by adipose tissue and conversion of glycogen into glucose-6-poshate in muscles. Obtained results present complex inter-relations that cannot be noticed by analyzing model balances alone, since global sensitivity analysis revealed important influences of specific parameters that are directly included into selected balances. In case of simulations with initial conditions according to Menu 2, global sensitivity coefficients for *v*_1_ and *v*_2_ were over the 50th percentile of calculated coefficients at *t*_f_ = 10 min (Figure 8a) and for *v*_19_ at *t*_f_ = 100 min (Figure 8c). Results showed that *v*_1_ and *v*_2_, reactions describing glucose-stimulated insulin production and insulin degradation, were mostly sensitive to *β*_6_, *β*_M_, *k*_10_ and *k*_14_, while *v*_19_ was mostly sensitive to changes in *β*_M_ and *k*_a_, which is similar for the simulations with initial conditions according to Menu 1 (0.157).

Furthermore, the sensitivities of the reactions included into plasma glucose, plasma insulin and plasma non-esterified fatty acids and plasma endogenous lipoprotein triglyceride balances were analyzed in detail. Hepatic lipid metabolism describes the plasma glucose change with glucose flux to plasma from the liver (*v*_4_), glucose flux from plasma to the liver (*v*_5_), insulin-stimulated glucose transport between plasma and muscle (*v*_25_) and glucose transport between plasma and adipose tissue (*v*_42_). For all four listed reactions, with both initial conditions, changes in control were noticed over the time frame. For example, *v*_4_ for both menus was mostly sensitive to k_22_, *β*_L_ and k_11_ at *t*_f_ = 10 min (Figure 7a and Figure 8a), while at *t*_f_ = 50, 100, 250 and 500 min, *β*_6_, *β*_M_, *k*_10_ and *k*_14_ (Figure 7b,e and Figure 8b,e) became the most important parameters. Results also showed that the highest global sensitivities for *v*_5_, *v*_25_ and *v*_42_ were obtained for *β*_L_ and *k*_11_. Insulin concentration change was described by glucose-stimulated insulin production (*v*_1_) and insulin degradation (*v*_2_). Gradually, both reactions with both indicial concentrations were mostly sensitive to *β*_6_, *β*_M_, *k*_10_ and *k*_14_. Plasma free fatty acid concentration was specified by free fatty acid uptake from plasma into muscle (*v*_33_), free fatty acid transport from plasma to the liver (*v*_17_), uptake of plasma free fatty acids into adipose free fatty acids (*v*_40_), release of adipose triglycerides to plasma free fatty acids (insulin-inhibited) (*v*_37_) and adipose tissue uptake of exogenous triglycerides from plasma (*v*_45_). Global sensitivity analysis showed that for both menus, the highest sensitivities for *v*_33_, *v*_17_, *v*_40_ and *v*_45_ were obtained for *β*_L_ and *k*_11_ at all times point (Figure 7 and Figure 8). However, it is also important to mention that values of sensitivities for *v*_33_, *v*_17_ and *v*_40_ decrease over time, while those for *v*_45_ were constant. Furthermore, endogenous plasma triglyceride concentration was modeled, taking into account the secretion of triglycerides from the liver (*v*_24_), export of triglycerides from the secretory pool to plasma (*v*_23_), liver uptake of triglycerides as free fatty acids (*v*_18_), uptake of plasma endogenous triglycerides into muscle free fatty acids (*v*_34_) and uptake of plasma endogenous triglycerides into adipose free fatty acids (*v*_39_). Obtained results showed that all reactions except *v*_23_ were mostly sensitive to changes in values of *β*_L_ and *k*_11_ for both menus and at all time intervals.

This study examined the influence of input parameter uncertainty on the variance of metabolic model predictions. The majority of the propagated uncertainty is due to the interactions of numerous factors rather than being linear from one parameter to one result as previously presented by Quang et al. [51].

## 4. Conclusions

This study looked at the overall impact of input parameter uncertainty on the metabolic model’s prediction variance. Instead of being linear from one parameter to one answer, the preponderance of the uncertainty that is spread results from the interactions of several other components. The obtained findings demonstrated that the model control varied depending on the various beginning concentrations as well as how the model control changed over time.

## Figures and Tables

**Table 1:** Designed meal plans with different proportions of macronutrients with an emphasis on the carbohydrates and fats.

	Meal	Components with Corresponding Masses
Menu 1	Breakfast	2 egg yolks (30 g), bacon (50 g) and gouda cheese (80 g) prepared in olive oil (5 g)
	Lunch	Salmon (100 g) prepared in olive oil (10 g) and green salad (100 g) with flax seeds (10 g) and olive oil (5 g)
	Snack	Handful of walnuts (30 g)
	Dinner	Chicken (120 g) prepared with cooking cream (150 mL) and broccoli (100 g) with olive oil (5 g)
Menu 2	Breakfast	Cornflakes (80 g) with 2.8% m.f. yogurt (200 mL) and a cup of chamomile tea with sugar (5 g)
	Lunch	Plate of vegetable soup (200 mL), chicken breast (200 g) prepared in olive oil (10 g) with couscous (50 g), tomato salad (100 g) with flax seeds (5 g), 2 slices of graham bread and a glass of orange juice (200 mL)
	Snack	Banana (100 g) and a handful of almonds (30 g)
	Dinner	Tuna steak (100 g) with potatoes (100 g) prepared with olive oil (10 g) and a cup of apple compote (200 mL)
Menu 3	Breakfast	2 egg yolks (30 g) and bacon (50 g) prepared in olive oil (5 g) and a slice of bread (25 g)
	Lunch	Plate of vegetable soup (200 mL), tuna steak (150 g) with potatoes (100 g) and Swiss chard prepared (100 g) in olive oil (10 g)
	Snack	3.2% m.f. yogurt (150 mL) and mixed nuts (40 g)
	Dinner	Chicken (100 g), rice (60 g) with vegetable salad (150 g) and olive oil (15 g)
Menu 4	Breakfast	2 slices of graham bread (50 g) with butter (15 g) and honey (20 g) and chamomile tea (200 mL) with sugar (5 g)
	Lunch	Plate of vegetable soup (200 mL), beef stew (150 g) prepared with olive oil (10 g) with pasta (80 g) and vegetable salad (100 g) with olive oil (5 g)
	Snack	Sliced apple (100 g) with peanut butter (10 g)
	Dinner	Salmon (100 g) prepared in olive oil (5 g) with bulgur (50 g), vegetable salad (100 g) with olive oil (5 g) and a glass of orange juice (200 mL)

**Table 2:** List of hepatic lipid metabolism model balances proposed by Pratt et al. [35].

Variable	Balance	Initial Conditions
plasma insulin	$\frac{dI}{dt}=k_{11}+k_{22}\cdot \mathrm{erf}\left(\frac{G_{B}-v}{cc}\right)-k_{d}\cdot I$	60 pmol/L
liver glucose	$\alpha_{L}\cdot \frac{dG_{L}}{dt}=S_{G}\left(t\right)-k_{gl}\cdot G_{L}+k_{gl2}\cdot G_{B}-\frac{v_{LG}\cdot G_{L}}{k_{LG}+G_{L}}-\frac{v_{LH}\cdot G_{L}}{k_{LH}+G_{L}}\cdot \left(\frac{1}{1+k_{rep}\cdot P_{L}}\right)\cdot \;k_{61}\cdot P_{L}$	8 mmol/L
liver glucose-6-phospahte	$\alpha_{L}\cdot \frac{dP_{L}}{dt}=-\frac{1}{2}\cdot k_{yl}\cdot I\cdot P_{L}\cdot \left(1+tanh\cdot \left(\frac{l_{max}-Y_{L}}{c_{0}}\right)\right)+\frac{\beta_{L}}{1+k_{p6}\cdot I}\cdot \left(\frac{Y_{L}}{Y_{L}+y_{0}}\right)-\;k_{p}\cdot I\cdot P_{L}+k_{gp}\cdot L_{A}+\frac{\beta_{6}\cdot R_{L}}{1+k_{p6}\cdot I}+\frac{v_{LG}\cdot G_{L}}{k_{LG}+G_{L}}+\frac{v_{LH}\cdot G_{L}}{k_{LH}+G_{L}}\cdot \left(\frac{1}{1+k_{rep}\cdot P_{L}}\right)-k_{61}\cdot P_{L}$	2.06 mmol/L
liver glycogen	$\alpha_{L}\cdot \frac{dY_{L}}{dt}=\frac{1}{2}\cdot k_{yl}\cdot I\cdot P_{L}\cdot \left(1+tanh\cdot \left(\frac{l_{max}-Y_{L}}{c_{0}}\right)\right)-\frac{\beta_{L}}{1+k_{dl}\cdot I}\cdot \left(\frac{Y_{L}}{Y_{L}+y_{0}}\right)$	50 mmol/L
liver pyruvate	$\alpha_{L}\cdot \frac{R_{L}}{dt}=k_{pp}\cdot R_{M}+\mu_{B}+k_{p}\cdot I\cdot P_{L}-\frac{\beta_{6}}{1+k_{p6}\cdot I}\cdot R_{L}-k_{al}\cdot I\cdot R_{L}+\mu_{B}$	0.37 mmol/L
free fatty acids in liver	$\alpha_{L}\cdot \frac{dA_{L}}{dt}=3\cdot k_{cl}\cdot T_{CB}+k_{bl}\cdot A_{B}+3\cdot k_{r}\cdot T_{LB}+k_{al}\cdot I\cdot R_{L}-\frac{3\cdot v_{6}\cdot A_{L}}{k_{6}+A_{L}}+\;\frac{3\cdot v_{10}\cdot T_{L}}{k_{10}+T_{L}}-\frac{3\cdot v_{8}\cdot A_{L}}{1+k_{5}\cdot I}-\frac{k_{7}\cdot A_{L}}{1+k_{5}\cdot I}$	0.57 mmol/L
triacylglycerides secretory pool in liver	$\alpha_{L}\cdot \frac{dS_{L}}{dt}=\frac{v_{6}\cdot A_{L}}{k_{6}+A_{L}}-k_{9a}\cdot S_{L}$	0.0149 mmol/L
triacylglycerol storage pool in liver	$\alpha_{L}\cdot \frac{dT_{L}}{dt}=\frac{v_{8}\cdot A_{L}}{k_{8}+A_{L}}-\left(k_{12}\cdot tanh\left(\frac{v_{12}-I}{k_{13}}\right)+k_{14}\right)\cdot \frac{v_{9}\cdot T_{L}}{k_{9}+T_{L}}-\frac{v_{10}\cdot T_{L}}{k_{10}+T_{L}}$	40 mmol/L
glucose in muscles	$\alpha_{L}\cdot \frac{dG_{M}}{dt}=\left(1+k_{gi}\cdot I\right)\cdot \left(k_{gm}\cdot G_{B}-k_{gm2}\cdot G_{M}\right)-\frac{v_{MH}\cdot G_{M}}{k_{MH}+G_{M}}\cdot \left(\frac{1}{1+k_{rep}\cdot P_{M}}\right)$	0.5 mmol/L
glycogen in muscle	$\alpha_{L}\cdot \frac{dY_{M}}{dt}=\frac{1}{2}\cdot k_{ym}\cdot I\cdot P_{M}\cdot \left(1+tanh\cdot \left(\frac{m_{max}-Y_{M}}{c_{0}}\right)\right)-\frac{\beta_{M}}{1+k_{dy}\cdot I}\cdot \left(\frac{Y_{M}}{Y_{M}+y_{0}}\right)$	20 mmol/L
glucose-6-posphate in muscle	$\alpha_{L}\cdot \frac{dP_{M}}{dt}=\frac{v_{MH}\cdot G_{M}}{k_{MH}+G_{M}}\cdot \left(\frac{1}{1+k_{rep}\cdot P_{M}}\right)-\frac{1}{2}\cdot k_{ym}\cdot I\cdot P_{M}\cdot \;\left(1+tanh\left(\frac{m_{max}-Y_{M}}{c_{0}}\right)\right)+\frac{\beta_{M}}{1+k_{dy}\cdot I}\cdot \left(\frac{Y_{M}}{Y_{M}+y_{0}}\right)-k_{6p}\cdot I\cdot P_{M}$	0.133 mmol/L
pyruvate in muscle	$\alpha_{L}\cdot \frac{R_{M}}{dt}=k_{6p}\cdot I\cdot P_{M}-k_{pp}\cdot R_{M}-\mu_{3}\cdot R_{M}\cdot I\cdot P$	0.009 mmol/L
free fatty acids in muscle	$\alpha_{L}\cdot \frac{A_{M}}{dt}=-3m_{s}\cdot I\cdot A_{M}+3m_{e}+3k_{cm}\cdot T_{CB}+k_{bm}\cdot A_{B}+3k_{t}\cdot T_{LB}-\mu_{4}\cdot A_{M}\cdot P$	0.53 mmol/L
triacylglycerides in muscle	$\alpha_{L}\cdot \frac{dT_{M}}{dt}=m_{s}\cdot I\cdot A_{m}-m_{e}$	14.8 mmol/L
AMP in muscles	$\frac{dP}{dt}=\mu_{amp}-\mu_{4}\cdot A_{M}\cdot P-\mu_{3}\cdot R_{M}\cdot I\cdot P$	0 mmol/L
adipose triacylglycerides	$\alpha_{L}\cdot \frac{dT_{A}}{dt}=k_{aa}\cdot I\cdot A_{A}\cdot G_{A}-\frac{\beta_{f}}{1+k_{ft}\cdot I^{2}}$	500 mmol/L
adipose free fatty acids	$\alpha_{L}\cdot \frac{dA_{A}}{dt}=-3\cdot k_{aa}\cdot I\cdot A_{A}\cdot G_{A}+3\cdot k_{a}\cdot \left(1+k_{ai}\cdot I\right)\cdot T_{CB}+3\cdot k_{ba}\cdot T_{LB}+k_{na}\cdot A_{B}$	0.57 mmol/L
adipose glycerol	$\alpha_{L}\cdot \frac{dL_{A}}{dt}=\frac{\beta_{f}}{1+k_{ft}\cdot I^{2}}-k_{gp}\cdot L_{A}$	0.17 mmol/L
adipose glucose	$\alpha_{L}\cdot \frac{dG_{A}}{dt}=d_{ba}\cdot \left(1+k_{ga}\cdot I\right)\cdot \left(G_{B}\cdot G_{A}\right)-k_{aa}\cdot I\cdot A_{A}\cdot G_{A}$	2.53 mmol/L
exogenous plasma triacylglycerides	$\frac{dT_{CB}}{dt}=S_{F}\left(t\right)-k_{cm}\cdot T_{CB}-k_{cl}\cdot T_{CB}-k_{a}\cdot \left(1+k_{ai}\cdot I\right)\cdot T_{CB}$	0 mmol/L
plasma free fatty acids	$\frac{A_{B}}{dt}=-k_{bm}\cdot A_{B}-k_{bl}\cdot A_{B}-k_{na}\cdot A_{B}+\frac{3\cdot \beta_{f}}{1+k_{ft}\cdot I}+3\cdot k_{a}$	0.5 mmol/L
endogenous plasma triacylglycerides	$\frac{dT_{LB}}{dt}=F\left(I\right)\cdot \frac{v_{9}\cdot T_{L}}{k_{9}+T_{L}}+k_{9a}\cdot S_{L}-k_{r}\cdot T_{LB}-k_{t}\cdot T_{LB}-k_{ba}\cdot T_{LB}$	Table 4
plasma glucose	$\frac{dG_{B}}{dt}=k_{gl}\cdot G_{L}-k_{gl2}\cdot G_{B}-\left(1+k_{gi}\cdot I\right)\cdot \left(k_{gm}\cdot G_{B}-k_{gm2}\cdot G_{M}\right)-d_{ba}\cdot \left(1+k_{ga}\cdot I\right)\cdot \left(G_{B}-G_{A}\right)-\mu_{1}$	Table 4

**Table 3:** List of hepatic lipid metabolism model parameters proposed by Pratt et al. [35].

	Parameter	Value
1.	*α*_A_ (adipose tissue volume)	15.60 L
2.	*α*_L_ (liver tissue volume)	1.60 L
3.	*α*_M_ (skeletal muscle volume)	26.4 L
4.	*β*_6_ (rate of liver de novo lipogenesis from pyruvate)	31.6 L/min
6.	*β*_f_ (adipose release of triacylglycerides to non-esterified fatty acids)	0.117 mmol/min
7.	*β*_L_ (liver glycogenolysis)	12 L/min
8.	*β*_m_ (muscle glycogenolysis)	82.5 L/min
9.	*µ*_amp_ (national adenosine monophosphate/adenosine diphosphate creation rate)	1.8
10.	*µ*_b_ (lactate production by red blood cells)	0.133 mmol/min
11.	*µ*_e_ (muscle triglyceride breakdown to free fatty acids)	0.420 mmol/min
12.	*µ*_s_ (muscle free fatty acid esterification to triglycerides)	7.19 × 10^6^ L mmol/min
13.	*µ*_1_ (plasma glucose usage)	0.588 mmol/min
14.	*µ*_3_ (muscle glucose-6-phospahte usage)	7.839 × 10^7^ L mmol/min
15.	*µ*_4_ (muscle free fatty acid usage)	100 L/min
16.	*c*_0_ (small parameters)	0.1 mmol/L
17.	*cc* (range of glucose concentrations over which excess insulin secretion occurs)	2.5 mmol/L
18.	*d*_ba_ (adipose uptake of glucose)	0.3 mmol/min
19.	*k*_10_ (affinity for hydrolysis of triglycerides to secretory pool)	0.625 mmol/L
20.	*k*_11_ (basal insulin secretion rate)	48 mmol/min
21.	*k*_12_ (increased fraction of very low-density lipoprotein 1 secretion by insulin)	0.2
22.	*k*_13_ (rate at which insulin modifies the fraction of very low-density lipoprotein 1 to very low-density lipoprotein 2 secretion)	15 mmol/L
23.	*k*_14_ (basal very low-density lipoprotein 1 secretion fraction)	0.6
24.	*k*_22_ (excess insulin secretion rate due to glucose stimulation)	48 mmol/min
25.	*k*_5_ (flux control coefficient for insulin inhibition of free fatty acid oxidation)	8.23 × 10^7^/mmol
26.	*k*_6_ (affinity for very low-density lipoprotein 2 triglyceride secretion through secretory pathway)	0.3 mmol/L
27.	*k*_61_ (liver glucose dephosphorylation rate)	4 L/min
28.	*k*_6p_ (muscle glucose-6-phospahte to pyruvate conversion rate)	6.56 × 10^8^ L^2^/mmol min
29.	*k*_7_ (maximum rate of free fatty acid oxidation)	0.759 L/min
30.	*k*_8_ (affinity for esterification of free fatty acids to triglycerides)	0.625 mmol/L
31.	*k*_9_ (affinity of additional bulk lipidation)	43.583 mmol/L
32.	*k*_9a_ (release of very low-density lipoproteins from secretory pathway)	1 L/min
33.	*k*_a_ (adipose free fatty acid uptake of chylomicron triglycerides, insulin independent)	0.1497 L/min
34.	*k*_aa_ (adipose free fatty acid esterification to triglycerides)	3.11 × 10^5^ L^2^/mmol min
35.	*k*_ai_ (adipose free fatty acid uptake of chylomicron triglycerides, insulin dependent)	2.08 × 10^6^ 1/mmol
36.	*k*_al_ (pyruvate to acetyl coenzyme A conversion rate)	0.00002 L^2^/mmol min
37.	*k*_ba_ (adipose uptake of endogenous lipoprotein triglycerides)	0.0104 L/min
38.	*k*_bl_ (liver uptake of plasma non-esterified fatty acids)	0.156 L/min
39.	*k*_bm_ (muscle uptake of plasma non-esterified fatty acids)	0.226 L/min
40.	*k*_cl_ (liver free fatty acid uptake of chylomicron triglycerides)	0.0075 L/min
41.	*k*_cm_ (muscle free fatty acid uptake of chylomicron triglycerides)	0.0449 L/min
42.	*k*_d_ (insulin degradation rate)	1.733 × 10^14^ L/mmol
43.	*k*_dl_ (liver glycogenolysis; insulin-inhibited rate)	3.5 × 18^8^ mmol/L
44.	*k*_dy_ (muscle glycogenolysis; insulin-inhibited rate)	4 × 10^8^ L/mmol
45.	*k*_ft_ (adipose release of triglyceride to non-esterified fatty acids; insulin-inhibited rate)	1.67 × 10^14^ L/mmol
46.	*k*_ga_ (rate of glucose diffusion between plasma and adipose mediated by glucose 4 transporters)	1.67 × 10^6^
47.	*k*_gi_ (glucose diffusion between plasma and muscles, insulin-mediated)	2.632 × 10^8^ mmol/L
48.	*k*_gl_ (plasma glucose diffusion rate to liver)	0.9277 mmol/L
49.	*k*_gl2_ (live glucose diffusion rate to blood)	0.396 mmol/L
50.	*k*_gm_ (plasma glucose diffusion rate to muscle)	0.0380 mmol/L
51.	*k*_gm2_ (muscle glucose diffusion rate to plasma)	0.0380 mmol/L
52.	*k*_gp_ (glucose-6-phospahte uptake from adipose glycerol)	0.311 L/min
53.	*k*_lp_ (rate of plasma triglyceride uptake by adipose tissue)	0.25
54.	*k*_LG_ (Michaelis–Menten constant of glucokinase in liver)	8.95 mmol/L
55.	*k*_LH_ (Michaelis–Menten constant of hexokinase in liver)	0.0115 mmol/L
56.	*k*_MH_ (Michaelis–Menten constant of hexokinase in muscle)	8.98 mmol/L
57.	*k*_na_ (rate of plasma free fatty acid uptake into adipose free fatty acids)	0.0697 L/min
58.	*k*_p_ (rate of insulin-mediated glucose-6-phoshate to pyruvate)	1.41 × 10^7^ mmol/L
59.	*k*_p6_ (constant of pyruvate conversion to glucose-6-phospate)	6.56 × 10^8^ L^2^/mmol min
60.	*k*_pp_ (rate of muscle pyruvate transport to liver)	0.5
61.	*k*_r_ (rate of endogenously derived lipoprotein triglycerides by liver as free fatty acids)	0.00058 mmol
62.	*k*_rep_ (glucose-6-phospahte inhibition constant of hexokinase in muscle)	2.98 mmol/L
63.	*k*_t_ (uptake rate of plasma endogenous triglycerides into muscle free fatty acids)	0.00348 mmol/L
64.	*k*_yl_ (rate of the glycogen synthesis stimulated by insulin)	1.28 × 10^6^
65.	*k*_ym_ (glycogen synthesis rate)	21.3641 mmol/L
66.	*l*_max_ (maximum glycogen store of liver)	400 mmol
67.	*m*_max_ (maximum glycogen concentration)	100 mmol
68.	*v* (rate of glycogen transport)	7 mmol
69.	*v*_10_ (rate of triglyceride storage conversion to free fatty acids)	0.1 mmol/min
70.	*v*_12_ (constant in triglyceride release into plasma)	40 mmol L^−1^
71.	*v*_6_ (rate of liver free fatty acid input to secretory pool)	0.0158 mmol/L
72.	*v*_8_ (rate of free fatty acid input to storage pool)	0.333 mmol/min
73.	*v*_9_ (rate of triglyceride release into plasma))	0.0159 mmol/L
74.	*v*_LG_ (maximum rate of glucokinase in liver)	14.3 mmol/min
76.	*v*_LH_ (maximum rate of hexokinase in liver)	5.57 mmol/min
76.	*v*_MH_ (muscle hexokinase maximum rate)	54.288 mmol/min
77.	*y*_0_ (range of liver glycogen concentration over which the release drops to zero)	0.1
78.	*α*_G_ (rate of glucose change in diet)	2 mmol
79.	*α*_F_ (rate of fructose change in diet)	2 mmol/L
80.	*m*_s_ (insulin-dependent rate of skeletal muscle storage of free fatty acids in the form of triglycerides)	0.8
81.	*m*_e_ (rate of skeletal muscle triglyceride breakdown to free fatty acids)	0.9

**Table 4:** Initial concentrations for the hepatic lipid metabolism model simulation based on developed menus.

	Menu 1	Menu 2	Menu 3	Menu 4
breakfast	*G*_B0_ = 1.5 mmol/L *T*_LB0_ = 23 mmol/L	*G*_B0_ = 44.7 mmol/L *T*_LB0_ = 1.6 mmol/L	*G*_B0_ = 7.7 mmol/L *T*_LB0_ = 16.1 mmol/L	*G*_B0_ = 23.6 mmol/L *T*_LB0_ = 4.7 mmol/L
lunch	*G*_B0_ = 3.2 mmol/L *T*_LB0_ = 9.1 mmol/L	*G*_B0_ = 49.8 mmol/L *T*_LB0_ = 9.5 mmol/L	*G*_B0_ = 14.0 mmol/L *T*_LB0_ = 7.7 mmol/L	*G*_B0_ = 43.2 mmol/L *T*_LB0_ = 14.5 mmol/L
snack	*G*_B0_ = 2.3 mmol/L *T*_LB0_ = 5.4 mmol/L	*G*_B0_ = 16.3 mmol/L *T*_LB0_ = 4.3 mmol/L	*G*_B0_ = 8.3 mmol/L *T*_LB0_ = 9.0 mmol/L	*G*_B0_ = 13.5 mmol/L *T*_LB0_ = 1.6 mmol/L
dinner	*G*_B0_ = 7.0 mmol/L *T*_LB0_ = 12.4 mmol/L	*G*_B0_ = 21.7 mmol/L *T*_LB0_ = 4.2 mmol/L	*G*_B0_ = 26.6 mmol/L *T*_LB0_ = 5.1 mmol/L	*G*_B0_ = 30.8 mmol/L *T*_LB0_ = 5.8 mmol/L

**Table 5:** Energy and macronutrient content (carbohydrates and fats) per meals according to meal plans with daily proportion of saturated fatty acids (SFA).

	Meal	Carbohydrates/g	Fats/g	SFA/%	Energy/kcal
Menu 1	Breakfast	2.7	82.4		859.2
	Lunch	5.7	32.6		408.6
	Snack	4.1	19.5		209.9
	Dinner	12.6	44.4		584.0
	**Per day**	**25.1**	**178.9**	**37.0**	**2061.7**
Menu 2	Breakfast	80.4	5.9		421.9
	Lunch	89.7	33.9		904.7
	Snack	29.3	15.3		284.1
	Dinner	39.1	15.1		395.1
	**Per day**	**238.5**	**70.2**	**2.7**	**2005.8**
Menu 3	Breakfast	13.9	57.7		612.9
	Lunch	25.2	27.7		517.3
	Snack	14.9	32.4		403.6
	Dinner	47.9	18.2		461.8
	**Per day**	**101.9**	**136.0**	**27.1**	**1995.6**
Menu 4	Breakfast	42.5	16.7		343.9
	Lunch	77.8	52.0		971.2
	Snack	24.3	5.6		164.4
	Dinner	55.4	20.8		533.6
	**Per day**	**200**	**95.1**	**9.8**	**2013.1**

## Data Availability

Not applicable.
